# Associations between Force-Time Related Single-Leg Counter Movement Jump Variables, Agility, and Linear Sprint in Competitive Youth Male Basketball Players

**DOI:** 10.3390/children10030427

**Published:** 2023-02-22

**Authors:** Ömer Pamuk, Yücel Makaracı, Levent Ceylan, Hamza Küçük, Tuba Kızılet, Tülay Ceylan, Erdi Kaya

**Affiliations:** 1 Department of Coaching Education, Faculty of Sports Sciences, Karamanoğlu Mehmetbey University, 70200 Karaman, Turkey; 2 Department of Coaching Education, Faculty of Sports Sciences, Sivas Cumhuriyet University, 58140 Sivas, Turkey; 3 Department of Physical Education and Sports Teaching, Yasar Doğu Faculty of Sport Sciences, Ondokuz Mayıs University, 55270 Samsun, Turkey; 4 Department of Coaching Education, Faculty of Sports Sciences, Marmara University, 34815 İstanbul, Turkey; 5 Department of Physical Education and Sports, Institute of Health Science, Sivas Cumhuriyet University, 58140 Sivas, Turkey; 6 Department of Recreation, Faculty of Sports Sciences, Akdeniz University, 07058 Antalya, Turkey

**Keywords:** adolescent, change of direction, speed, vertical jump, limb asymmetry, unilateral strength, team sports

## Abstract

Background: Previous research has reported a strong relationship between vertical jumping, sprinting, and agility, as a reflection of lower-limb power. Unilateral analysis of this relationship has not yet been explored. This study primarily investigated the associations between single-leg countermovement jump (CMJ), sprint, and agility performances in youth basketball players. Methods: Thirty-five male basketball players from the youth category (age 15.06 ± 2.62 years, n = 32 right-limb dominant; n = 3 left-limb dominant) performed single-leg CMJ, 20 m sprint, and T-drill agility tests over two sessions. Force–time-related performance variables were measured using a single-leg CMJ test on a Kistler force plate. Results: Significant moderate to large negative correlations were observed between single-leg CMJ variables, 20 m sprint, and T-drill agility, except for mean force for both dominant and non-dominant leg measures (r = −0.384 to −0.705). Mean power and mean force were correlated with the physical characteristics of the athletes for both legs (r = −0.389 to −0.843). Flight time and jump height were identified as the best predictor variables for both sprint and agility time in the stepwise model (R^2^ = 0.608 to 0.660). No statistical inter-limb differences were found during the single-leg CMJ test (*p* > 0.05). Conclusions: The study findings suggest that youth basketball players with greater single-leg jump output most likely have better sprint and agility performances. Thus, trainers and athletic performance coaches may include unilateral limb exercises in their training programs to enhance lower-limb explosive performance and reduce limb asymmetries.

## 1. Introduction

Anaerobic-based explosive movements related to basketball can be categorized into high-intensity activities requiring jumping, change of direction (COD), running speed, and acceleration/deceleration [1,2,3]. Such movement patterns in explosive form require a high level of lower-extremity power [4,5]. These movement patterns are connected to the development of strength, sprint, and agility. In fact, such common motor skills in the sport have similar biomechanical features and power outputs. The execution of explosive and dynamic movements affects the transition from attacking to defending (and vice versa) in basketball [6,7]. Therefore, the reflection of lower-limb performance could affect basketball-specific abilities (e.g., fast dribbling, shooting, lay-up, block, and rebound) [8]. Furthermore, previous studies indicated a correlation between the agility and linear sprint performance of athletes from different competition levels [9,10,11] The performance level of explosive strength outputs, mainly in the enhancement of the vertical jump, are essential for basketball performance [12]. Vertical jump ability is also considered an assessment for kinematics and kinetics of the lower extremity, [13] and it can be measured in both unilateral and bilateral stance [14]. Optimum jumping performance is important for defensive and offensive tasks [15]. Most of the vertical jump tasks are performed unilaterally by a single leg [16,17]. Taken together, a possible inter-limb asymmetry could negatively impact athletic performance.

Several performance tests are used to characterize lower-limb functional ability and quantify limb asymmetries by measuring unilateral strength [18]. Single-leg countermovement (CMJ) represents a reliable measure of limb asymmetry and sport-specific movement patterns [19,20]. Variations in force–time-related jump performances (i.e., power, force, acceleration, and time) between dominant and non-dominant legs in single-leg CMJ tasks are indicative of functional strength asymmetries [21]. However, lower limb-related strength and balance outputs demonstrate bilateral asymmetries [22]. Additionally, competitive athletes are characterized by increased bilateral asymmetries following various forms of unilateral injuries [23]. Indeed, previous studies show that a bilateral asymmetry threshold of >15% indicates potential injury risk in healthy subjects, although this may be influenced by injury history and physical fitness [24]. Force plates are considered the “gold standard” in recording ground reaction force (GRF)-derived data [25]. Connected to this, lower extremity strength and possible asymmetries could be accurately assessed by CMJ performed on a force plate. However, no study has examined the GRF-derived single-leg CMJ performance and its relationship with sprint and agility in basketball players. Studying this possible relationship may be important to define force–time-related jumping parameters.

Short-distance sprint, COD, jumping, and technical abilities as well as body height and optimal body mass [26] should be analyzed together to understand overall basketball performance [27]. A few studies reported a correlation among lower-limb based explosive movements such as jump, sprint, and agility in basketball [4,10]. So, if a significant association is found between these movements and single-leg CMJ performance, the results may help practitioners and athletic trainers to focus on unilateral performance parameters to optimize sprint and agility, which are critically important for basketball performance [28,29,30]. Furthermore, the impact of the age factor on the aforementioned relationship remains unclear. Physical fitness during adolescence is essential for maintaining overall health and athletic performance [31]. Therefore, the GRF-derived single-leg CMJ parameters and their associations with agility and sprint in youth basketball players are unique points that should be investigated.

Considering the relationship between vertical jumping, sprinting, and agility, as a reflection of lower-limb power, a unilateral analysis in the dominant and non-dominant leg context will provide important data for further research. Thus, in line with the insights previously mentioned, the primary purpose of this study was to investigate the association of force–time-related single-leg CMJ performance with linear sprinting and agility in youth basketball players. Furthermore, we aimed to examine the lower-limb strength asymmetry during a unilateral task. Accordingly, the study hypotheses were established as follows: (1) Force–time-related single-leg CMJ performance variables would correlate with sprinting and agility; (2) force–time-related single-leg CMJ performance variables would correlate with physical characteristics of the athletes; (3) dominant and non-dominant leg single-leg CMJ performance variables would not significantly differ.

## 2. Materials and Methods

### 2.1. Participants

Study participants included 35 youth competitive male basketball players (age range 14–17 years; n = 32 right-limb dominant; n = 3 left-limb dominant) from a domestic basketball club in Turkey, and all were familiar with athletic testing procedures. The dominant limb of the participants was identified as the leg that would be used to kick a ball [32] and by a single-leg CMJ test (highest height indicating the dominant leg). The baseline characteristics of the study participants are presented in Table 1.

The criteria for inclusion in the study included being a member of the youth category team (<18 years old), regularly participating in team training sessions (physical and technical–tactical), and having no musculoskeletal injury. The exclusion criteria included reporting severe pain/discomfort during the test, having a lower-extremity injury, or having had surgery in the last six months.

### 2.2. Study Design and Procedures

To test the study hypothesis, a cross-sectional design was used by determining a representative sample group. All study measurements were conducted over two testing sessions on separate days at least 24 h apart during the pre-season period of the athletes. The athletes’ anthropometric data and single-leg CMJ measurements were tested during the first session, while the 20 m sprint and T-drill agility tests were conducted during the second session. All study measurements were performed at the training facility of the athletes, under the direct supervision of the study researchers, and were held from 12 to 2 pm.

The Kistler force plate (Kistler, Winterthur, Switzerland; type 9260AA6; natural frequency ≈ 400 Hz), which has a usable surface of 60 × 50 × 5 cm, placed on a flat and rigid floor, was used to collect the single-leg CMJ test data. The signals received from the force plate were transferred to a personal laptop computer (HP Probook 450 G6 with Core i7) through a data acquisition board (type 5691A; Winterthur, Switzerland; USB 2.0). The kinetic data were then gathered using Bioware Software and saved as a Microsoft Excel (Microsoft Corp., Redmond, WA, USA) document. The quantified test parameters referring to the single-leg CMJ were recorded utilizing Kistler’s Measurement, Analysis and Reporting Software (MARS) (Kistler MARS, S2P Ltd., Ljubljana, Slovenia), which is commercially viable [33]. The performance times for the 20 m sprint and T-drill agility tests were assessed on a standard basketball court using a portable wireless photocell system (Witty, Microgate, Bolzano, Italy) connected to an electronic timer.

A standardized warming-up protocol for each session prior to the athletic testing process was performed by the athletes. This protocol involves stretching/mobility exercises in major muscle groups belonging to the lower limbs and three sub-maximal vertical jumps for the single-leg CMJ test during the first session. The athletes also performed running/COD-based drills for the 20 m sprint and T-drill agility tests in the second session [10]. During the study measurements, athletes were required to wear their own running shoes in order to counteract the impacts of various athletic equipment. The participation of the athletes in moderate-to-high-intensity exercise (≥24 h) was restricted before each session.

### 2.3. Ethics

The study protocol was approved by the research ethics committee of Karamanoğlu Mehmetbey University, with ethical approval number 03-2022/59. Signed parental consent for the study was obtained from all subjects before beginning the investigation.

### 2.4. Measures

#### 2.4.1. Anthropometrics

The body height and body mass of the athletes were measured at the beginning of the first session using a stadiometer (Seca 220, Hamburg, Germany) and a Kistler force plate (just before single-leg CMJ testing), respectively.

#### 2.4.2. Single-Leg CMJ Test

**Figure 1:**
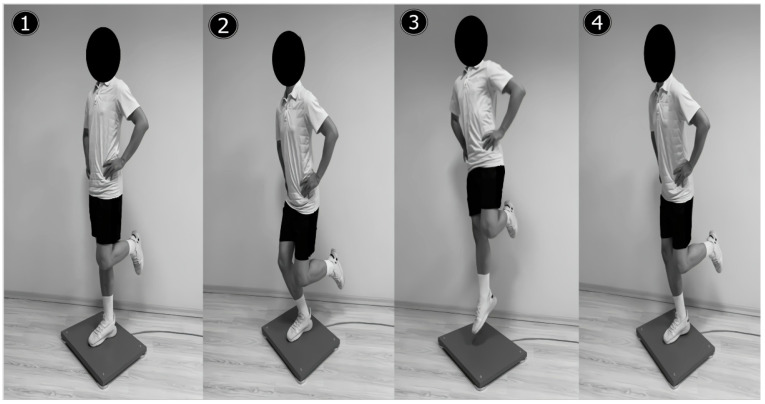
Phases of a single-leg countermovement jump on a force plate. (1) Standing (quiet), (2) braking, (3) take-off (flight), and (4) landing.

The athletes were instructed to step onto the center of the force plate, where they performed the single-leg CMJ test for both dominant and non-dominant legs, respectively. The test began with the dominant leg fully extended on the force plate (center point) and the opposite leg at hip level, with the knee joint at 90° flexion. To standardize the same test position for all participants during the trials, hands were placed on the hips. Subsequently, after performing a “counter-movement” reaching a self-determined depth (braking), the athlete was instructed to jump as high as possible (take-off). The test trial ended by landing on the plate with the tested leg following the take-off phase [34] (Figure 1). During the take-off phase, the athletes were not permitted to swing or move the opposite leg in any form and this situation was cautiously monitored by a study researcher. A repetition performed in accordance with the specified test procedure was accepted as valid by the force plate software, and the average of a total of three successful repetitions was used for further analysis. A rest period of 30 s separated the repetitions to prevent fatigue.

Force–time-related single-leg CMJ performance variables obtained from the MARS, including jump height (calculated from take-off velocity) (m), relative maximum power (W/kg), mean power (W), mean force (N), mean velocity (m/s), acceleration (m/s^2^), vertical take-off velocity (m/s), and flight time (s), were used for further analyses. 

#### 2.4.3. T-Drill Agility Test

**Figure 2:**
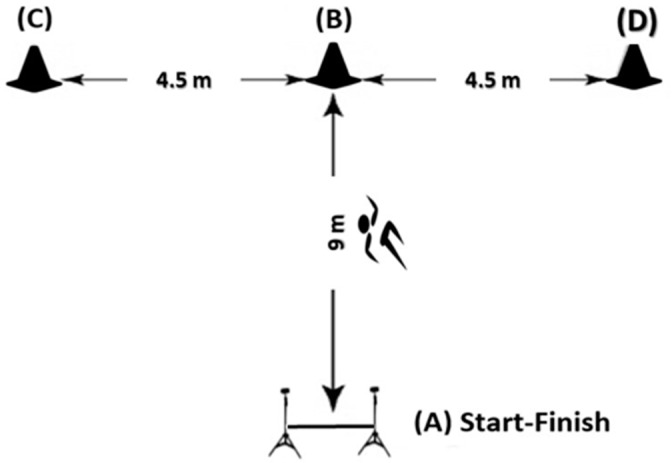
T-drill test.

The T-drill agility test includes multidirectional (lateral, forward, and backward) running and CODs (see Figure 2). The test procedures were conducted as previously described by Makaracı and Soslu [10]. In brief, the test started with a sprint passing through the photocell gate (point A) by activating the timer automatically. Then the athlete was required to sprint forward 9 m and touched the cone on the ground (point B). At that point, the athlete side-shuffled to the left, without crossing his feet, to another cone 4.5 m away (point C). The athlete then suddenly shuffled 9 m in the opposite direction (to the right) to touch the cone with the right hand (point D). Finally, the athlete side-shuffled back to the cone in the middle (point C) and backpedaled to the starting point (point A). The hand that was on the same side as the running/shuffle direction was used to touch the cones (i.e., the right hand when shuffling to the right). Each athlete performed the test twice, with a 2 min rest between the trials, and the best performance was recorded in seconds.

#### 2.4.4. 20-Meter Sprint Test

**Figure 3:**
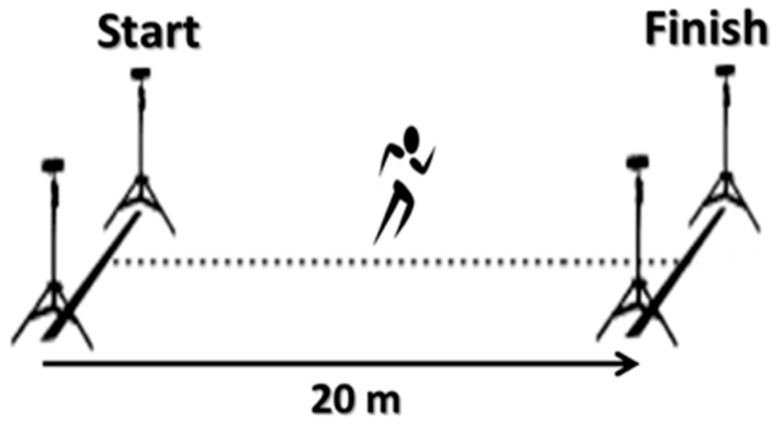
20 m sprint test.

The linear sprint was assessed over a 20 m protocol, which has been deemed to be a reliable method for basketball players. The test began at the starting point in an upright stride stance with the preferred leg forward, 0.5 m before the first photocell gate (see Figure 3). The athletes were instructed to perform all the sprint trials with a maximal effort. Each athlete performed two sprint trials, with a minimum of 2 min of rest but no longer than 3 min. The best performance of the two trials (recorded in seconds) was used for subsequent analysis.

### 2.5. Data Analysis

An a priori power analysis was performed using an alpha value of 0.05 and an 80% (β) confidence interval (effect size = 0.70; critical t  =  1.9965; df  =  66) with G*Power 3 statistical software; the necessary sample size would require at least 34 participants for the present study. An SPSS for Windows 25.0 (SPSS Inc., Chicago, IL, USA) package program was used in the data analysis. The participants’ baseline characteristics were expressed as mean and standard deviation. A Levene’s test was conducted for the assumption of the equality of variance. The Shapiro–Wilk test was used to determine data normality, and the data were found to be non-normally distributed. The relationships between the single-leg CMJ (dominant and non-dominant legs), sprinting, agility, and physical variables were measured by Spearman’s correlation. The magnitude of correlations was based on the following criteria: ≤0.1, trivial; 0.1–0.3, small; 0.3–0.5, moderate; 0.5–0.7, large; 0.7–0.9, very large; and 0.9–1.0, almost perfect [35]. Stepwise multiple regression analyses were used to determine which single-leg CMJ variables (best predictor model) could predict 20 m sprint and T-drill agility scores for both limbs, and these variables were reported. Coefficients of determination (R^2^), both crude and adjusted, were used to interpret the meaningfulness of the relationships. The single-leg CMJ performance difference between the dominant and non-dominant legs was determined by the Mann–Whitney U test. Effect sizes were reported based on Cohen’s recommendations, where 0.2–0.49 is a small effect, 0.5–0.79 is a moderate effect, and ≥0.8 is a large effect [36]. The significance of the effects was assumed at *p* < 0.05 for all the analyses.

## 3. Results

The results of Spearman’s correlation between the single-leg CMJ variables and 20 m sprint/T-drill agility scores are presented in Table 2.

Significant moderate to very large negative correlations were observed between single-leg CMJ variables, 20 m sprint, and T-drill agility, except for mean force for the dominant leg (r-range from −0.384 to −0.705). Similarly, significant moderate to large negative correlations were observed between single-leg CMJ variables, 20 m sprint, and T-drill agility, except for mean force for the non-dominant leg (r-range from −0.419 to −0.643).

The results of Spearman’s correlation for the single-leg CMJ and physical characteristics (body mass, body height, and body mass index) are presented in Table 3. 

Significant moderate to very large positive correlations were observed between mean power–mean force and all physical characteristics of the athletes for both dominant and non-dominant leg measures (r-range from 0.389 to 0.843).

The stepwise multiple regression analysis results of single-leg CMJ variables that influenced the 20 m sprint and T-drill agility scores are examined in Table 4.

Table 4 demonstrates that the best predictor model for 20 m sprint and T-drill agility times included only flight time for the dominant leg measures. Flight time explained 64% of the variance (R^2^ = 0.636) for the 20 m sprint and 61% of the variance (R^2^ = 0.608) for T-drill agility. In the non-dominant leg measures, the best predictor model for the 20 m sprint score included only jump height (R^2^ = 0.637). Mean velocity (R^2^ = 0.597) and jump height (R^2^ = 0.660) were the included variables for the T-drill agility score.

Mann–Whitney U test results for the comparison of dominant and non-dominant leg CMJ variables with 95% confidence intervals are presented in Table 5.

No statistical inter-limb differences were observed for the athletes during the single-leg CMJ test, although there was a tendency in favor of dominant legs in all parameters (*p* > 0.05).

## 4. Discussion

Considering the relationship among lower-limb performance, including a fast stretch–shortening cycle and explosive mechanisms [37], an analysis from a unilateral perspective will provide crucial data for force–time-related CMJ measures. To the best of our knowledge, this study is the first that examines the GRF-derived single-leg CMJ performance and its relationship with sprint and agility in basketball players. More specifically, the main aim of the present study was to investigate the associations of force–time-related single-leg CMJ performance with linear sprint and agility in youth basketball players. The second purpose was to reveal inter-limb differences during the single-leg CMJ. Consistent with the study hypothesis, our results showed negative correlations between the single-leg CMJ, 20 m sprint, and T-drill agility for both dominant and non-dominant leg measures in male youth basketball players. We also found positive correlations between mean power–mean force and all physical characteristics of the athletes for both limbs. Flight time (dominant leg) and jump height (non-dominant leg) were identified as the predictor variables for both sprint and agility time in the stepwise model. However, we did not observe an inter-limb difference for single-leg CMJ variables.

Jumping ability and performance are among the primary factors for basketball-specific defensive and offensive movements [38]. Simenz et al. [39] reported that jumping performance is related to motoric characteristics, such as power, agility, and speed, in athletes. It was also suggested that jumping ability could be associated with physical fitness features. Nikolaidis et al. [27] indicated that body mass is related to jumping performance in male basketball players (9–12 years old), similar to our findings. On the other hand, many forms of jumping movement during competition occur unilaterally [40]. Correspondingly, the single-leg CMJ is a commonly used test protocol for lower-limb explosive power, representing a stronger indicator of the capacity of each limb [41,42]. Our results showed a negative significant correlation between the single-leg CMJ, sprinting, and agility in both dominant and non-dominant leg measures (see Table 2). The force–time-related data we obtained from the single-leg CMJ test provide valuable findings for this correlation. While the CMJ performance is usually expressed by jump height, it largely depends on various jump-based variables. In our study, we observed that, along with the jump height, relative maximal power and mean velocity parameters were strongly associated with 20 m sprint and T-drill agility scores in both legs (*p* < 0.001; r-range from −0.548 to −0.705). We also observed that flight time and jump height were the best-fitting equations for sprint and agility (Table 4). Therefore, these parameters may be considered an indicator of lower-limb explosive power in youth male basketball players. Based on these findings, it can be suggested that focusing jumping exercises on unilateral form can be a useful strategy to enhance lower-limb explosive movements (sprint and agility) in basketball players during the adolescent period.

Previous research confirmed the relationships among vertical jumping, sprinting, and agility in athletes or trained adults. Stojanovic et al. [43] previously reported a high negative correlation between the CMJ and repetitive sprinting in basketball players (r = −0.74). Alemdaroğlu [4] also indicated a relationship between sprinting, agility, and jump height during SJ and CMJ tests in professional male basketball players (r-range between −0.48 and −0.76). In different studies, negative correlations between jump performance and linear sprint were also observed in healthy, physically active adults [39,44,45]. However, given that the data were collected unidirectionally (i.e., jump height, flight time), it is highly likely that this result was not fully explored. We have found only one study investigating this relationship from a unilateral point of view. In this study, Yanci et al. [46] noted significant moderate correlations between non-dominant leg CMJ, 15 m sprint and 505 agility in football players. However, we used single-leg CMJ measures by using GRF-derived data in the present study. The unique parameters, such as relative maximum power, acceleration, and vertical take-off velocity, were calculated as force–time-related data. Similar parameters have been recently used in a study conducted by Makaracı and Soslu [10]. Their results demonstrated that the jumping performance in different tasks (CMJ, squat, and drop jump) correlated with sprinting and agility in male basketball players of different competitive groups. Even though this finding confirms the relationships between force–time-related jump variables and sprint/agility, the jumping performance is evaluated bilaterally. Nonetheless, it can be stated that this research partially supported our findings. Overall, considering the number of studies examining the aforementioned correlation in basketball players is quite limited, our novel study findings can make some important contributions to further investigations.

Even though the main purpose of our study was to examine the relationship between single-leg CMJ performance and sprinting and agility, the recognition of a potential inter-limb difference should be considered a crucial detail for the study results. The asymmetries of >10% may adversely affect jumping (i.e., jump height) [47] and COD performance [48]. Hence, the single-leg CMJ not only represents lower-limb power but also shows inter-limb asymmetries [49]. Moreover, the assessments of inter-limb differences based on force–time-related variables are considered a valid and practically accessible method for athletes from different sports [50]. The presence of possible inter-limb asymmetries is justified by the influence that CMJ-based asymmetry may have on athletic performance [51]. Moreover, the inter-limb differences in athletes have been established mostly by comparing dominant and non-dominant limbs [52]. Our findings revealed no statistical inter-limb differences during the unilateral CMJ test, although there was a trend in favor of dominant legs in all parameters (see mean scores in Table 3). The trend in asymmetry favoring the dominant limb may be explained by the realization of explosive movements in basketball, such as sprinting, jumping, and COD, with the more intensive use of the dominant leg [53]. Some similar findings were observed in previous research for the direction of limb asymmetries. Fort-Vanmeerhaeghe et al. [41] reported stronger dominant leg measures in terms of CMJ height, which is similar to our results (the tendency in asymmetry to favor the dominant limb). Similarly, Kozinc and Šarabon [54] reported similar findings in relation to the agreement on the direction of the asymmetries within the use of various tests involving the single-leg CMJ in youth volleyball players. Furthermore, the determined partial bilateral balance in our study can be considered a factor supporting the correlation findings discussed in detail above. Summing up, our findings may be beneficial for coaches to maintain lower-limb explosive performance and also to reduce asymmetry-related injury risk.

This study has limitations. First, although the single-leg CMJ was preferred as a reflection of lower-limb power in our study, different test procedures specific to the lower limbs can be used to examine potential correlations with sprint and agility. Second, we did not measure the lower-limb muscle power (i.e., electromyographic activity) of the athletes during the single-leg CMJ test as the explosive movements could be associated with muscular power. Third, the athletes in this study were all youth basketball players, and thus the results may not be generalizable to athletes from different sports and competitive levels. Finally, the study results should be taken with caution because the correlations do not imply a cause-and-effect association.

## 5. Conclusions

A negative relationship was revealed between force–time-related single-leg CMJ, sprint, and agility performances in adolescent basketball players. Mean power and mean force related to single-leg CMJ performance were correlated with physical characteristics of the athletes. We also found no statistical inter-limb differences during the single-leg CMJ test, although there was a tendency in favor of dominant legs. The present study findings, therefore, suggest that single-leg CMJ, short-distance speed, and COD ability share common biomechanical and physiological characteristics. Our study also suggests that youth basketball players with greater single-leg-jump output most likely have better sprint and agility performances. Thus, trainers and athletic performance coaches must pay attention to unilateral limb exercises in order to enhance lower-limb explosive performance and reduce leg asymmetries.

## Figures and Tables

**Table 1:** Baseline characteristics of the athletes (Mean ± SD).

Variables	n = 35
Age (years)	15.06 ± 2.62
Body mass (kg)	76.46 ± 18.69
Body height (cm)	180.17 ± 31.02
Body mass index (kg·m^−2^)	23.46 ± 2.54
Sports experience (years)	4.67 ± 1.05
20 m sprint (s)	3.41 ± 0.26
T-drill agility (s)	12.33 ± 1.04

**Table 2:** Correlation coefficients between the single-leg CMJ and sprint/agility scores for dominant and non-dominant legs.

		JH (m)	RMP (W/kg)	AC (m/sn^2^)	VTOV (m/s)	MP (W)	MF (N)	MV (m/s)	FT (s)
DL	20 m sprint (s)	−0.705	−0.671	−0.419	−0.630	−0.487	−0.166	−0.626	−0.666
		0.000 ***	0.000 ***	0.014 **	0.000 ***	0.004 **	0.348	0.000 ***	0.000 ***
	T-Drill agility (s)	−0.685	−0.646	−0.384	−0.645	−0.480	−0.147	−0.634	−0.669
		0.000 ***	0.000 ***	0.023 *	0.000 ***	0.004 **	0.407	0.000 ***	0.000 ***
NDL	20 m sprint (s)	−0.643	−0.577	−0.458	−0.496	−0.494	−0.189	−0.603	−0.492
		0.000 ***	0.000 ***	0.007 **	0.003 **	0.003 **	0.285	0.000 ***	0.003 **
	T-Drill agility (s)	−0.610	−0.548	−0.469	−0.420	−0.484	−0.158	−0.578	−0.419
		0.000 ***	0.001 **	0.005 **	0.013 *	0.004 **	0.373	0.000 ***	0.014 *

Notes. DL: dominant leg; NDL: non-dominant leg; JH: jump height; RMP: relative maximum power; AC: acceleration; VTOV: vertical take-off velocity; MP: mean power; MF: mean force; MV: mean velocity; FT: flight time (s). * *p* < 0.05; ** *p* < 0.01; *** *p* < 0.001.

**Table 3:** Correlation coefficients between the single-leg CMJ and physical characteristics of the athletes for dominant and non-dominant legs.

		JH (m)	RMP (W/kg)	AC (m/sn^2^)	VTOV (m/s)	MP (W)	MF (N)	MV (m/s)	FT (s)
DL	Body mass (kg)	−0.131	−0.031	−0.088	−0.174	0.471	0.843	−0.180	−0.159
		0.460	0.863	0.619	0.324	0.005 **	0.000 ***	0.309	0.368
	Body height (cm)	0.015	0.087	0.194	0.081	0.464	0.566	0.028	0.077
		0.935	0.626	0.271	0.647	0.006 **	0.000 ***	0.876	0.665
	BMI (kg·m^−2^)	−0.121	−0.038	−0.064	−0.163	0.389	0.694	−0.154	−0.160
		0.497	0.833	0.720	0.358	0.023 *	0.000 ***	0.386	0.365
NDL	Body mass (kg)	0.119	0.130	0.082	0.160	0.466	0.793	−0.041	0.138
		0.504	0.462	0.644	0.367	0.006 **	0.000 ***	0.819	0.437
	Body height (cm)	0.134	0.166	0.146	0.112	0.464	0.633	0.202	0.085
		0.450	0.349	0.411	0.529	0.006 **	0.000 ***	0.252	0.631
	BMI (kg·m^−2^)	0.162	0.155	0.165	0.139	0.425	0.664	−0.028	0.115
		0.361	0.381	0.352	0.433	0.012 *	0.000 ***	0.877	0.518

Notes. DL: dominant leg; NDL: non-dominant leg; BMI: body mass index; JH: jump height; RMP: relative maximum power; AC: acceleration; VTOV: vertical take-off velocity; MP: mean power; MF: mean force; MV: mean velocity; FT: flight time (s). * *p* < 0.05; ** *p* < 0.01; *** *p* < 0.001.

**Table 4:** Stepwise multiple regression analysis results of single-leg CMJ variables that influenced the 20 m sprint and T-drill agility scores for dominant and non-dominant legs.

	Leg	Model	Unstandardized Coefficients		CB	t	*p*	R^2^ (Adjusted R^2^)
			B	SE				
20 m sprint (s)	DL	(Constant)	4.457	0.228	−0.636	19.562	0.000	0.636 (0.404)
		FT (s)	−3.239	0.694		−4.657	0.000	
	NDL	(Constant)	3.884	0.108	−0.637	36.117	0.000	0.637 (0.406)
		JH (m)	−4.387	0.938		−4.675	0.000	
T-drill agility (s)	DL	(Constant)	16.350	0.938	−0.608	17.422	0.000	0.608 (0.370)
		FT (s)	−12.418	2.864		−4.335	0.000	
	NDL	(Constant)	15.066	0.666	−0.597	22.608	0.000	0.597 (0.356)
		MV (m/s)	−2.771	0.659		−4.208	0.000	
		(Constant)	15.129	0.634	−0.358	23.850	0.000	0.660 (0.436)
		JH (m)	−9.873	4.709		−2.097	0.044	

Notes. DL: dominant leg; NDL: non-dominant leg; JH: jump height; MV: mean velocity; FT: flight time; SE: standard error; CB: coefficients beta.

**Table 5:** Comparison of dominant and non-dominant leg CMJ variables.

Parameters	Leg	n	Mean	SD	95% CI	*p*	ES
Jump height (m)	DL	35	0.12	0.06	0.10 to 0.14	0.534	0.19
	NDL		0.11	0.04	0.09 to 0.12		
Relative maximum power (W/kg)	DL	35	26.68	7.56	24.27 to 29.28	0.874	0.03
	NDL		26.45	9.14	23.42 to 29.48		
Acceleration (m/sn^2^)	DL	35	1.77	0.95	1.45 to 2.08	0.304	0.22
	NDL		1.54	1.14	1.16 to 1.92		
Vertical take-off velocity (m/s)	DL	35	1.55	0.42	1.41 to 1.69	0.133	0.30
	NDL		1.43	0.38	1.30 to 1.55		
Mean power (W)	DL	35	1069.50	383.45	942.46 to 1196.53	0.545	0.10
	NDL		1030.29	391.48	900.59 to 1159.98		
Mean force (N)	DL	35	1113.15	219.58	1040.40 to 1185.90	0.842	0.09
	NDL		1092.12	219.40	1019.43 to 1164.80		
Mean velocity (m/s)	DL	35	1.04	0.24	0.96 to 1.12	0.356	0.07
	NDL		1,02	0.30	0.92 to 1.12		
Flight time (s)	DL	35	0.32	0.09	0.29 to 0.35	0.133	0,35
	NDL		0.29	0.08	0.26 to 0.32		

Notes. DL: dominant leg; NDL; non-dominant leg; CI: confidence interval; ES: effect size; Cohen’s d effect size, where 0.2–0.49 is a small effect, 0.5–0.79 is a moderate effect, and ≥0.8 is a large effect.

## Data Availability

The data used to support the findings of the current study are available from the corresponding author upon request.
